# Phylogenetic relationships of *Vepris* (Rutaceae) inferred from chloroplast, nuclear, and morphological data

**DOI:** 10.1371/journal.pone.0172708

**Published:** 2017-03-08

**Authors:** Cynthia M. Morton

**Affiliations:** Pittsburgh Parks Conservancy, Pittsburgh, PA; Institute of Botany, CHINA

## Abstract

The tribe Toddalieae Hook. F. (Rutaceae) has been controversial since its inception by Bentham and Hooker. The nine taxa examined, *Acronychia* J.R. & G.Foster, *Diphasia* Pierre, *Diphasiopsis* Mendonca, *Fagaropsis* Mildbr.ex. Siebenl., *Oricia* Pierre, *Teclea* Delile, *Toddaliopsis* Engl., *Toddalia* Juss. and *Vepris* Comm. ex. A. Juss, have been recognized under the tribe Toddalieae or Tribes Acronychia, Phellodendron and Toddalia. More recently *Araliopsis* Engl., *Diphasia*, *Diphasiopsis*, *Oricia*, *Teclea*, and *Toddaliopsis* have been incorporated into the genus *Vepris*, while *Toddalia* and *Fagaropsis* have continued to be recognized as closely related. For this study, sequence data of one non-coding chloroplast region (*trnL-F)* and one nuclear region (ITS) and various morphological characters, based on Mziray’s taxonomic studies were examined to try to elucidate these relationships. This study found that the taxa *Diphasia*, *Diphasiopsis*, *Oricia*, *Teclea*, *Toddaliopsis*, *Vepris*, *Toddalia eugeniifolia* Engl. and *Toddalia glomerata* F. Hoffm. form a monophyletic group. Due to the amount of intrageneric and intraspecific variation, species delimitations were difficult to determine; however, these genera should be united into *Vepris*. The analyses also confirmed that *Toddalia asiatica* (L.) Lam., *Zanthoxylon* sp. and *Fagaropsis angolensis* (Engl.) H.M. Gardner are the closest relatives to this group.

## Introduction

Generic circumscriptions within the tribe Toddalieae Hook. F. (Rutaceae) in Africa has been controversial since its inception. Toddalieae is one of seven tribes given to the Rutaceae by Bentham and Hooker (1867) [1]. This tribe along with the tribe Aurantieae were grouped together in (1867) based on ovary and fruit similarities. Engler (1896) [2] rearranged the family to include six subfamilies and ten tribes. To separate the tribes, he mainly used the number of carpels (2–5 in the Toddalieae). Later he (1917) [3] described a new genus, *Humblotiodendron* Engl., under the Toddalieae; however, Perrier (1948) [4] reduced it to synonymy in *Vepris*. Verdoorn (1926) [5] revised the African Toddalieae and recognized seven genera but the available material for study was inadequate and the key to the genera ignored the fact that that many plants are dioecious so male specimens cannot be identified. Since then more material has been collected and additional taxa have been described, allowing the overlap and character-inconsistency between genera to become evident. Engler, (1931 [6]) increased the number of tribes to eleven and subdivided the Tolddalieae into six subtribes (Table 1). A chemosystematic review of the family by da Silva et al. (1988 [7]) proposed major changes in the Englerian scheme. In this proposed scheme Rutoideae and Toddaliodeae are united, the subtribal groups are dispensed with, and most of the taxa are brought together with the Australasian genus *Acronychia* J.R. Forst. & G. Forst. under the tribe Acronychia while *Toddalia* and *Fagaropsis* are classified in separate tribes. Kokwaro, (1982) synonymized *Tecleopsis* into *Vepris* but still recognized *Toddalia*, *Toddaliopsis*, *Diphasiopsis*, *Teclea*, *Diphasia* and *Fagaropsis*. Kokwaro (1982) [8] did not recognize a higher classification system. Hall and Waterman, (1979) [9] synonymized *Oriciopsis* Engl. into *Vepris*. The most recent taxonomic studies in the Toddalieae was completed by Mziray (1992) [10], who, based on morphology, recognized three genera, rather than the original nine; the three genera are *Vepris*, *Toddalia* and *Fagaropsis*. The largest genus is *Vepris*, which incorporates *Araliopsis* Engl., *Diphasia*, *Diphasiopsis*, *Oricia*, *Teclea*, and *Toddaliopsis*. Accordingly, the nomenclature used in this paper is that of Mziray (1992) [10] (Table 1).

**Table 1:** Position of African *Vepris* members (bold face) in selected earlier classifications. Only the relevant parts of the schemes of Engler (1931) and de Silva et al. (1988) are reproduced. Figures in brackets after names of taxa indicate the approximate number of species included.

Engler (1931)	da Silva et al. (1988) informal proposal	Mziray (1992)
Subfam. TODDALIOIDEAE	Subfam. RUTOIDEAE (s. lat.)	TODDALIEAE (tribe)
TODDALIEAE-tribe	ZANTHOXYLUM-tribe	***Vepris*** (c. 91)
		(= ***Araliopsis* (2)**,
		= ***Diphasia* (6)**,
		***Diphasiopsis*** Mendonca **(2),**
		***Oricia* (6)**,
		***Teclea* (c. 25)**, and
		***Toddaliopsis* (2)**)
Phellodendrinae (subtribe)	TODDALIA-tribe	***Todalia* (1)**
*Phellodendron* Rupr.	***Toddalia***	***Fagaropsis* (c. 4)**
*Clausenopsis* Engl. (= ***Fagaropsis*** Mildr.)	PHELLODENDRON-tribe	
Sohnreyiinae (1) (subtribe)	*Phellodendron*	
*Sohnreyia* K. Krause	***Fagaropsis***	
Pteleinae (4) (subtribe)	PENTACERAS-tribe	
*Helietta* Tul.	EUODIA-tribe	
*Taravalia* Greene	ACRONYCHIA-tribe	
*Balfourodendron* Mello ex Oliv.	***Acronychia***	
*Ptelea* L.	***Vepris***	
Oriciinae (subtribe)	***Araliopsis***	
***Oricia*** Pierre	***Oriciopsis*** (= *Vepris)*	
***Diphasia*** Pierre	***Oricia***	
Toddaliinae (subtribe)	***Diphasia***	
***Araliopsis*** Kurz	***Teclea***	
*Sargentia* S. Watson (= *Casimiroa greggii* (S.Watson) FChiang)	LUNASIA-tribe	
*Casimiroa* La Llave	PTELEA-tribe	
***Vepris*** Juss.	CUSPARIA-tribe	
***Toddalia*** Juss	CASIMIROA-tribe	
***Toddaliopsis*** Engl.	DECATROPSIS-tribe	
***Oriciopsis*** Engl. (= *Vepris glaberrima* (Engl.) J.B. Hall)	AMYRIS-tribe	
*Humblotiodendron* Engl. (= *Vepris madagascarica* (Baill.) H. Perrier)	BORONIA-tribe	
***Acronychia*** J.R. Forst. & G. Forst.	DIOSMA-tribe	
*Bauerella* Schindl. (= *Sarcomelicope* Engl.)	CHOISYA-tribe	
*Halfordia* F. Muell.	RUTA-tribe	
*Hortia* Vand.	DICTAMNUS-tribe	
*Skimmia* Thunb.		
Amyridinae (subtribe)		
*Amyris* P. Browne		
***Teclea*** Delile		
Stauranthus		

Subfamilial phylogenetic analyses were completed for the Rutaceae by Chase et al. (1999) [11], Groppo et al. (2008) [12], Poon et al. (2007) [13], and Morton and Telmer (2014) [14], using evidence from *rbcL*, *atpB*, *rps16*, *trnL-trnF*, *trnL-F*, *xdh*, and ITS sequence variation. None of the above authors, included taxa from *Araliopsis*, *Diphasia*, *Diphasiopsis*, *Oricia*, *Teclea*, or *Toddaliopsis*. Only two unidentified species of *Vepris* were included by Groppo et al. (2008) [12], so, their relationship to each other and to other taxa of Rutaceae based on molecular techniques needs to be examined in order to assess the degree of congruence with morphological characters.

The goals of this study are (1) to evaluate the genera within *Vepris* as recognized by Mziray (1992) [10]; (2) to test the monophyly of the *Vepris* and to identify the closest relatives; (3) to examine the relationship based on congruence of morphology and molecular characters.

## Methods

For this study, one non-coding chloroplast region (*trnL-trnF*), as well as, one nuclear region (ITS) and various morphological characters were selected. The *trnL-trnF* region consists of the *trnL* intron and the *trnL-trnF* intergenic spacer [15]. ITS consists of three genes that code for the *18S*, *5*.*8S* and *26S* ribosomal subunits. The three genes are separated by two internal transcribed spacers, ITS1 between *18S* and *5*.*8S* and ITS2 between *5*.*8S* and *26S*. In addition to the extensive use of the rapidly evolving ITS spacer sequences in phylogenetic studies at lower levels [16, 17], the sequences have also served to resolve intrafamilial relationships [18]. Morphological characters were taken from information in Mziray ([10], pages 43–45) on taxonomic studies in Toddalieae.

### Taxon sampling & DNA extraction

Vouchers for the 85 species used in this study along with the GenBank accession numbers are listed in the Table 2. Some specimens were collected using a National Geographic Society Grant in association with the University of Dar es Salaam (COSTECH and USD immigration permits were issued). The following taxa were not sequenced due to a lack of material: *Araliopsis* and *Oriciopsis*. The total genomic DNA was extracted from (0.5–1.0 g) fresh or dried leaf material. Leaves were ground with a mortar and pestle and subsequently treated with the DNEasy plant DNA extraction kit from Qiagen (Qiagen, Valencia, California, USA) following the manufacturer’s protocol. Alignments were made using the Sequencher software program (Gene Codes Corporation, Ann Arbor, MI), for each marker and also the broader *trnL-F* alignment with sampling across all Rutaceae subfamilies including Meliaceae and Simaroubaceae as outgroups.

**Table 2:** GenBank accession numbers for molecular data sets from one chloroplast marker (trnL) and one nuclear marker (ITS). Newly sequenced taxa for this study are in bold with voucher information. All remaining GenBank accession numbers are from previous studies, as indicated by footnotes. References indicated by superscript after number and papers listed below.

Species	Accession/Voucher	TrnL/TrnF	ITS1/ITS4
Acronychia pedunculata (L.) Miq		KJ158057^[^^37^^]^	
Acronychia vestita F. Muell.		KC42843^[^^37^^]^	
Adenandra uniflora (L.) Willd.		JX307298^[^^14^^]^	
Aegle marmelos (L.) Corrêa		AY29529^[^^30^^]^	
Atalantia ceylanica (Arn.) Oliver		AY295288^[^^30^^]^	
Balfourodendron riedelianum (Engl.) Engl		FJ716791^[^^36^^]^	
Bauerella simplicifolia (Engl) syn. Sarcomelicope simplicifolia (Engl.) Hartley		EU853766 ^[^^12^^]^	
Calodendrum capensis (L. f.) Thunb.		AF025511^[^^32^^]^	
Casimiroa tetrameria Millsp.		EU853782 ^[^^12^^]^	
Chloroxylon swietenia DC.		AY295276^[^^30^^]^	
Choisya dumosa var. mollis (Standl.) L.D.Benson		EU853784 ^[^^12^^]^	
Citropsis articulata (Willd. ex Spreng.) Swingle & M.Kellerm.	4935381, MO	KU193629	
Citropsis articulata (Willd. ex Spreng.) Swingle & M.Kellerm.	568514, MO	KU193630	KU193662
Citrus glauca (Lindl.) Burkill. syn. Eremocitrus glauca (Lindl.)		AY295293^[^^30^^]^	
Clausena anisata (Willd.) Hook.f. ex Benth.	529294, CM	KU193626	KU193659
Clausena anisata (Willd.) Hook.f. ex Benth.	417111, MO	KU193627	KU193660
Clausena anisata (Willd.) Hook.f. ex Benth.	568514, MO	KU193628	KU193662
Clausena excavata Burm. f.		EF126674^[^^29^^]^	
Cneorum pulverulentum Vent.		EU853787^[^^12^^]^	
Correa pulchella J. Mackay ex Sweet		EU853790^[^^12^^]^	
Chorilaena quercifolia Endl.		EU853785^[^^12^^]^	
Dictamnus albus L.		EU853793^[^^12^^]^	
Dictyoloma vandellianum A. Juss.		EU853793^[^^12^^]^	
Diphasia morogorensis Kokwaro	4959977, MO	KU193631	KU193663
Diphasiopsis fadenii Kokwaro	5993752, MO	KU193645	KU193677
Diplolaena dampieri Desf.		EU853794^[^^12^^]^	
Eriostemon angustifolius Paul G. Wilson		JX307299^[^^14^^]^	
Euodia hortensis J.R. Fort & G. Forst		HG002786^[^^35^^]^	
Fagaropsis angolensis (Engl.) H.M.Gardner	529288, CM.	KU193632	KU193664
Fagaropsis angolensis (Engl.) H.M.Gardner	529290, CM	KU193633	KU193665
Flindersia australis R. Br.		EF126677^[^^29^^]^	
Fortunella polyandra (Ridl.) Tanaka		AY295291^[^^30^^]^	
Glycosmis pentaphylla (Retz.) DC		AY295279^[^^30^^]^	
Halfordia kendack Guillaumin		EU853798^[^^12^^]^	
Harrisonia abyssinica (Oliv.)		EU721390^[^^34^^]^	
Harrisonia abyssinica (Oliv.)	178980, AU	KU193625	
Harrisonia abyssinica (Oliv.)		FR747904^[^^35^^]^	
Harrisonia abyssinica (Oliv.)			GU178980^[^^35^^]^
Helietta puberula R.E. Fr.		EU853799^[^^12^^]^	
Hortia superba Ducke		EU853804^[^^12^^]^	
Lunasia amara Blanco		EU853805^[^^12^^]^	
Melicope ternata J. R. Forst. & G. Forst.		EU853808^[^^12^^]^	
Murraya paniculata (L.) Jack		EU853810^[^^12^^]^	
Orixa japonica Thunb.		EF489254 ^[^^33^^]^	
Oricia swynnertonii Verdc.	3262937, MO		KU193658
Phebalium woombye Domin		JX307300^[^^14^^]^	
Phellodendron amurense Rupr.		DQ225993^[^^13^^]^	
Pilocarpus spicatus A. St.-Hil		EU853811^[^^12^^]^	
Poncirus trifoliate (L.) Raf.		AY295282^[^^30^^]^	
Ptaeroxylon obliquum (Thunb.) Radlk		EU853812^[^^12^^]^	
Ptelea trifoliate L.		FJ716780^[^^36^^]^	
Ravenia infelix Vell.		EU853814^[^^12^^]^	
Ruta graveolens L.		AY295275^[^^30^^]^	
Sarcomelicope simplicifolia (Endl.) T. G. Hartley		EU853816^[^^12^^]^	
Severinia buxifolia (Poir.) Ten. Syn. Atalantia buxifolia (Poir.) Oliv. ex Benth.		EU369566^[^^31^^]^	
Simaba bidwillii (Hook. f.) Feuillet		JX307326^[^^14^^]^	
Simaba cedron Planch.		EU853768^[^^12^^]^	
Skimmia japonica Thunb.		EU853819^[^^12^^]^	
Skimmia anquetilia N.P. Taylor & Airy Shaw		EF126698^[^^29^^]^	
Spathelia excelsa (K. Krause) R. S. Cowan & Brizicky		EU853820^[^^12^^]^	
Sweitenia macrophylla King		EF489262^[^^33^^]^	
Teclea amaniensis Engl. Syn. Vepris amaniensis (Engl.) Mziray	29281, CM	KU193634	KU193666
Teclea amaniensis Engl. Syn. Vepris amaniensis (Engl.) Mziray	Boyona, AF	KU193651	KU193683
Teclea amaniensis Engl. Syn. Vepris amaniensis (Engl.) Mziray	4244019, MO	KU193637	KU193669
Teclea hanangensis Kokwaro syn. Vepris hanangensis (Kokwaro) Mziray	5725823, MO	KU193640	KU193672
Teclea nobilis Delile syn. Vepris nobilis (Delile) Mziray	529291, CM	KY508614	KY508613
Teclea nobilis Delile syn. Vepris nobilis (Delile) Mziray	4598475, MO	KU193638	KU193670
Teclea simplicifolia (Engl.) I. Verd syn. Vepris simplicifolia (Engl.) Mziray	5345141, MO	KU193657	KU193689
Teclea simplicifolia (Engl.) I. Verd syn. Vepris simplicifolia (Engl.) Mziray	5902918, MO	KU193643	KU193675
Teclea simplicifolia (Engl.) I. Verd syn. Vepris simplicifolia (Engl.) Mziray	Butz, AF	KU193636	KU193668
Teclea trichocarpa (Engl.) Engl. Syn. Vepris trichocarpa (Engl.) Mziray	5341454, MO	KU193646	KU193678
Teclea trichocarpa (Engl.) Engl. Syn. Vepris trichocarpa (Engl.) Mziray	Salt, AF	KU193647	KU193679
Teclea trichocarpa (Engl.) Engl. Syn. Vepris trichocarpa (Engl.) Mziray	4998848, MO	KU193639	KU193671
Teclea trichocarpa (Engl.) Engl. Syn. Vepris trichocarpa (Engl.) Mziray	529279, MO	KU193635	KU193667
Tetradium ruticarpum (A. Juss.) T.G. Hartley		DQ225984^[^^13^^]^	
Toddalia asiatica (L.) Lam.	Minjasin, AF	KU193641	KU193673
Toddalia asiatica (L.) Lam.	6177836, MO	KU193642	KU193674
Toddalia eugeniifolia Engl.	5614653, MO	KU193652	KU193684
Toddalia glomerata F. Hoffm.	5693948, MO	KU193654	KU193686
Toddalia lanceolate Lam. Syn. Vepris undulata Verdoorn & C. A. Sm	5769420, MO	KU193653	KU193685
Toddaliopsis sansibarensis Engl. Syn. Vepris sansibarensis (Engl.) Mziray	5750529, MO	KU193649	KU193681
Toddaliopsis heterophylla (Engl.) Engl. Syn. Vepris heterophylla (Engl.) Letouzey	5518017, MO	KU193650	KU193682
Vepris stolzii I.Verd.	5315965, MO	KU193644	KU193676
Vepris stolzii I.Verd.	5700270, MO	KU193648	KU193680
Zanthoxylum chevalieri P.G.Waterman	6177214, MO	KU193655	KU193687
Zanthoxylum deremense (Engl.) Kokwaro	5301622, MO	KU193656	KU193688
Zanthoxylum fagara (L.) Sarg		EF126684^[^^29^^]^	
Zanthoxylum rhoifolium Lam.		EU853773^[^^12^^]^	

### trnL-trnF

The *trnL* intron and the *trnL-trnF* intergenic spacer for 80 (*Oricia* samples could not be amplified) species were amplified. PCR was performed using the universal primers trn-c, trn-d, trn-e, and trn-f as described by Taberlet et al. (1991) [15]. For some samples the entire *trnL* intron/*trnL-trnF* spacer region was amplified with trn-c and trn-f. In others, two separate amplifications were performed, one to amplify the *trnL* intron with trn-c and trn-d and the other to amplify the *trnL-trnF* spacer with trn-e and trn-f. The DNA fragment amplified using these primers is approximately 800bp long. The final PCR cocktail of 50 mL contained the following: 38 mL water, 5 mL 10% buffer, 3 mL Mg^+2^, 1 mL dNTPs, 0.25 mL *Taq* polymerase, and 0.5 mL of each primer along with 1 ul of DNA template for each reaction. PCR amplification used a 7-min denaturing step at 94°C followed by 30 cycles of denaturing for 1 min at 94°C, primer annealing for 1 min at 45°C, and elongation for 1 min at 72°C, with a final 7 -min elongation step at 72°C.

### ITS

The amplification of the ITS gene was performed successfully on 35 species using oligonucleotide primers ITS1/ITS4 [19] to acquire the entire region (one sample of *Citropsis* Swingle & Keller could not be amplified). The DNA fragment amplified using these two primers is approximately 800bp long and includes ITS1, ITS2 and the *5*.*8S* ribosomal gene. The basic mix contained the following: 38μl of H_2_O, 5μl of 10% Mg free buffer solution, 3–6 μl of 25mM MgCl_2_, 1μl of 10mM dNTPs, 0.5μl of each primer (10nM), and 0.25μl *Taq* DNA along with 1 μl of DNA template for each reaction. The thermal cycler was programmed to perform an initial 1 cycle of denaturation at 95°C for 2 min. followed by 24 cycles of 30 seconds at 55°C, 72°C for 1 min. 30 seconds and 95°C for 30 seconds. This was followed by a 10 min. extension at 72°C to allow completion of unfinished DNA strands.

### Cycle sequencing

The PCR products were cleaned using the QIAGEN QIAquick PCR purification kit (QIAGEN Inc., Chatsworth, California, USA) following the protocols provided by the manufacturer. Cleaned products were then directly sequenced using the ABI PRISM Dye Terminator Cycle Sequencing Ready Kit with AmpliTaq DNA Polymerase (Applied Biosystems Inc., Foster City, California, USA). Unincorporated dye terminators were removed using the QIAGEN DyeEx dye-terminator removal system (QIAGEN Inc.) following the manufacturer’s recommendations. Samples were then loaded into an ABI 3100 DNA Sequencer. The sequencing data was analyzed and edited using the Sequencher software program (Gene Codes Corporation, Ann Arbor, Michigan, USA).

### Morphological characters

Thirty-three characters are morphological. Seventeen characters were coded as unordered binary and 16 as multistate. All characters were variable. All analyses were conducted as stated in the analysis section. Character states of taxa were taken from Mziray ([10], pages 43–45).

### Phylogenetic analysis

Boundaries of the *trnL* intron, and the ITS nuclear gene were determined by comparison with sequences in Genbank. The following two alignment criteria and methodology were used: (1) when two or more gaps were not identical but overlapping, they were scored as two separate events and (2) phylogenetically informative indels (variable in two or more taxa) were scored as one event at the end of the data set. All DNA sequences reported in the analyses have been deposited in Genbank (Appendix 1).

All phylogenetic analyses employed maximum-parsimony with the heuristic search option in PAUP* 4.0b8 [20] with uninformative characters excluded. Searches were conducted with 1000 random-taxon-addition replicates with TBR branch swapping, steepest descent, and MulTrees selected with all characters and states weighted equally and unordered. All trees from the replicates were then swapped onto completion, all shortest trees were saved, and a strict consensus or majority rule tree was computed. Relative support for individual clades was estimated with the bootstrap method [21]. One thousand pseudoreplicates were performed with uninformative characters excluded. Ten random-taxon-addition heuristic searches for each pseudoreplicate were performed and all minimum-length trees were saved for each search. To reduce bootstrap search times, branches were collapsed if their minimum length was zero (“amb-“).

Unambiguous morphological state changes were identified by using a combined analysis and MacClade 4.0 [22].

Bayesian analyses were performed using MrBayes 3.1.1 [23] on the combined molecular datasets accessed through the CIPRES portal [24]. The substitution model for each DNA region was selected with MrModeltest 2.3 [25] under the Akaike information criterion (AIC). The parameters for the Bayesian analyses were as follows: nst = 6; rates = gamma; set autoclose = yes; mcmcp ngnen = 10000; samplefreq = 10; savebriens = yes and the first 25% of the trees were regarded as “burn in”. Branch lengths are averaged from the distribution of trees and the posterior probability values (BPP) for the branches reported [25].

For the likelihood analyses, the program MrModelltest 2.3 [25] was used to select the models of nucleotide evolution. Maximum likelihood trees were calculated using the web service for GARLI 2.1 ([26]., 2014; available at www.molecularevolution.org). A total of 1000 replicates were conducted using the combined datasets and using the GTR + I + G model.

To determine the combinability of the data sets, their data structures were compared using methods outlined by Mason-Gamer and Kellogg (1996) [27], who discussed various ways to assess conflict between data sets. In one method the combination of independent data sets is possible if the trees do not conflict or if conflict receives low bootstrap support. Therefore, each node on the independent trees is tested for congruence against the other. If the nodes do not contain conflicting information, they are congruent and the data sets are combinable. Where there are incongruent nodes, the bootstrap values for each node are examined. If the support is less than 70%, there is no hard conflict and the incongruence is interpreted as being due to chance. In this study the different data sets were analyzed independently and in combination to see how each data set changed or confirmed the tree topologies of each other and to adopt a hypothesis of phylogenetic relationships for the tribes and genera.

## Results

The inclusion of gap coding in all data sets containing molecular data resulted in more homoplasy and lack of resolution; therefore, gap coding was not used in the following results. Genbank sequences KU193 625-KU193689 were specifically generated for this study.

### Larger trnL-trnF family analysis

**Fig 1:**
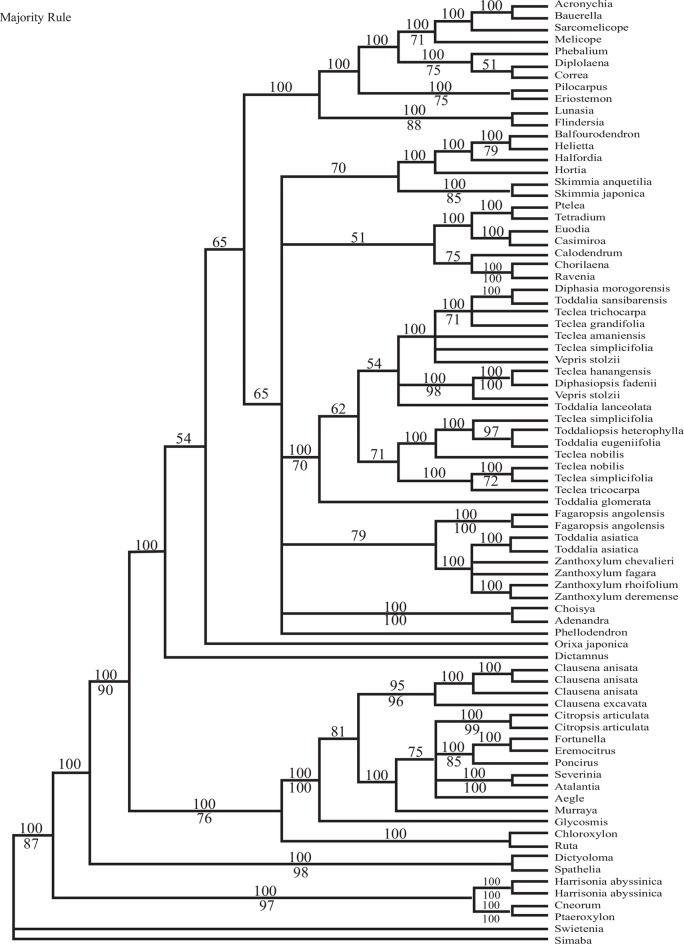
Majority rule tree of the expanded “trnL-trnF” data set using various genera of Rutaceae. Numbers below nodes are bootstrap values.

Multiple sequence alignment of 78 Rutaceae and two closely related taxa resulted in a data matrix of 1038 characters. No regions were excluded. Of the 1038 positions constituting the aligned *trnL-trnF* sequences, 690 (66%) were variable and 392 (38%) were parsimony-informative. The analysis recovered 390 equally optimal trees of 1281 steps (CI = 0.54, RI = 0.71; Fig 1).

*Vepris* is not monophyletic in the majority rule tree because *Toddalia eugeniifolia*, and *Toddalia glomerata* fall within the clade. *Diphasia*, *Diphasiopsis*, *Teclea*, *Toddalia*, *Toddaliopsis*, and *Vepris* do form a clade. Five clades form a polytomy with the above clade as follows: 1. ((*Balfourodendron* and *Helietta* BS 79%*)*, *Halfordia*, *Hortia*, *Skimmia*); 2. (((*Ravenia* and *Chorilaena*, BS 100%), *Calodendrum*) ((*Ptelea* and *Tetradium*) (*Euodia* and *Casimiroa*))); 3. ((*Toddalia* and *Zanthoxylon*), *Fagaropsis*); 4. *Choisya* and *Adenandra* (BS 100%); and 5. *Phellodendron*, followed by the remaining taxa.

### Combined molecular data using parsimony

Following the methods outlined by Mason-Gamer and Kellogg (1996) [27], the data sets were considered combinable. Within each gene analysis, *trnL-trnF*, and ITS, *Vepris* was not monophyletic and needs to be re-circumscribed. Among the molecular trees there was only one conflicting node with bootstrap support greater than 75% as follows: within the *trnL-trnF* two species of *Teclea simplicifolia* and *Teclea nobilis* (BS 84%) formed a clade whereas in the ITS two species of *Teclea simplicifolia* (BS 92%) formed a clade sister to a clade containing *Teclea nobilis*, *Teclea trichocarpa* and *Toddaliopsis heterophylla*. The conflict is in the position of *T*. *nobilis* which has no BS support in the ITS clade, therefore congruence exists between the data sets and a combined molecular analysis was completed.

**Fig 2:**
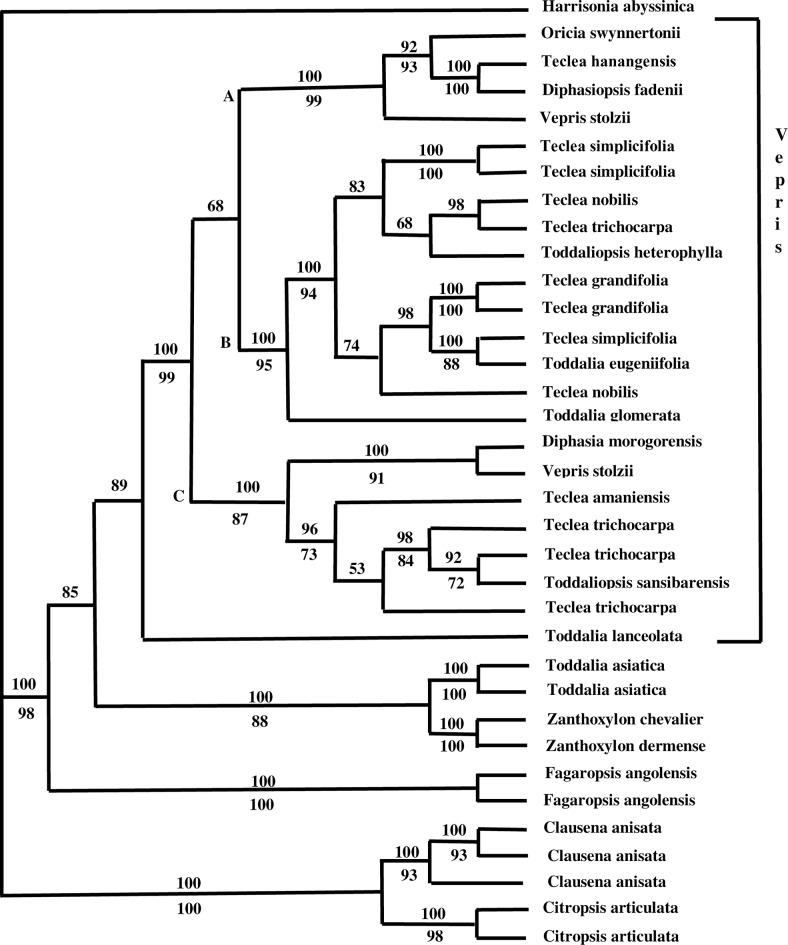
Majority rule tree (length = 1134 steps, CI = 0.64, RI = 0.80) obtained from all molecular data. Numbers below nodes are bootstrap values.

Multiple sequence alignment of the 35 Rutaceae samples resulted in a matrix of 1763 characters, of which 2% include at least one accession with a gap. Mean percentage G + C content is 47%. Of the 1763 positions constituting the aligned sequences, 682 (38.7%) were variable and 443 (25%) were parsimony informative. The analysis recovered 61 equally optimal trees of 1134 steps (CI = 0.64, RI = 0.80; Fig 2 majority rule tree).

*Vepris* consists of three clades labeled as A, B. and C. Clade A contains ((((*Teclea hanangensis* and *Diphasiopsis fadenii* BS 100%) *Oricia swynnertonii* BS 93%) and *Vepris stolzii*) (BS 99%)).

Clade B contains (2 species of *Teclea simplicifolia* BS 100% sister to (*Teclea nobilis* and *Teclea trichocarpa*) *Toddaliopsis hererophylla*) this clade is sister to ((2 species of *Teclea grandifolia* BS 100%) (*Teclea simplicifolia* and *Toddalia eugeniifolia* BS 88%) *Teclea nobilis*). *Toddalia glomerata* was at the base to the above clades. Clade C consists of ((((*Teclea trichocarpa* and *Toddaliopsis sansibarensis* BS 72%) *Teclea trichocarpa* BS 84%) *Teclea trichocarpa*) *Teclea amanuensis*) sister to ((*Diphasia morogroensis* and *Vepris stolzii* BS 91%) (BS 87%)). At the base of clades A, B and C is the taxon *Toddalia lanceolate* and then a clade containing two species of (*Toddalia asiatica* (BS 100%) sister to two species of *Zanthoxylon* (BS 100%) BS 88). At the base of this clade are two species of *Fagaropsis angolensis* (BS 100%) followed by a clade consisting of *Clausena anisata* and *Citropsis* articulate.

**Fig 3:**
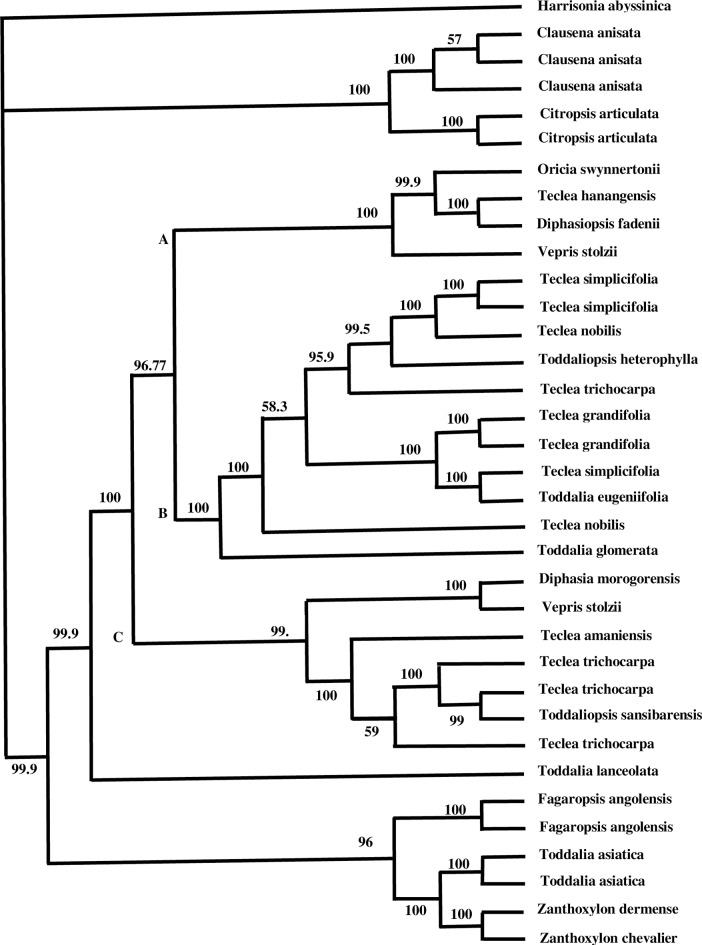
Bayesian majority rule consensus tree using molecular data. Numbers above the nodes are posterior probability values.

In the Bayesian combined analysis, the group of exemplars from *Vepris* were not monophyletic because *Toddalia eugeniifolia* and *Toddalia glomerata* is within the clade; Fig 3. In all but two branches, the posterior probability values were higher than 99%. The only difference between the majority rule tree and the Bayesian tree was in clade B. In the majority rule tree, positions of ((*Teclea nobilis*, *T*. *trichocarpa*) and *Toddaliopsis herterophylla*)) formed a non-supported clade whereas in Bayesian tree, these taxa formed a grade. In addition, in clade B, a sample of *Teclea nobilis* in the majority rule tree at the base of the *Teclea grandifolia*, *T*.*simplicifolia*, and *Toddalia eugenifolia* clade whereas in the Bayesian tree *Teclea nobilis* was at the base of both the clades. None of the majority rule clades had support over 50% therefore there was no conflict.

**Fig 4:**
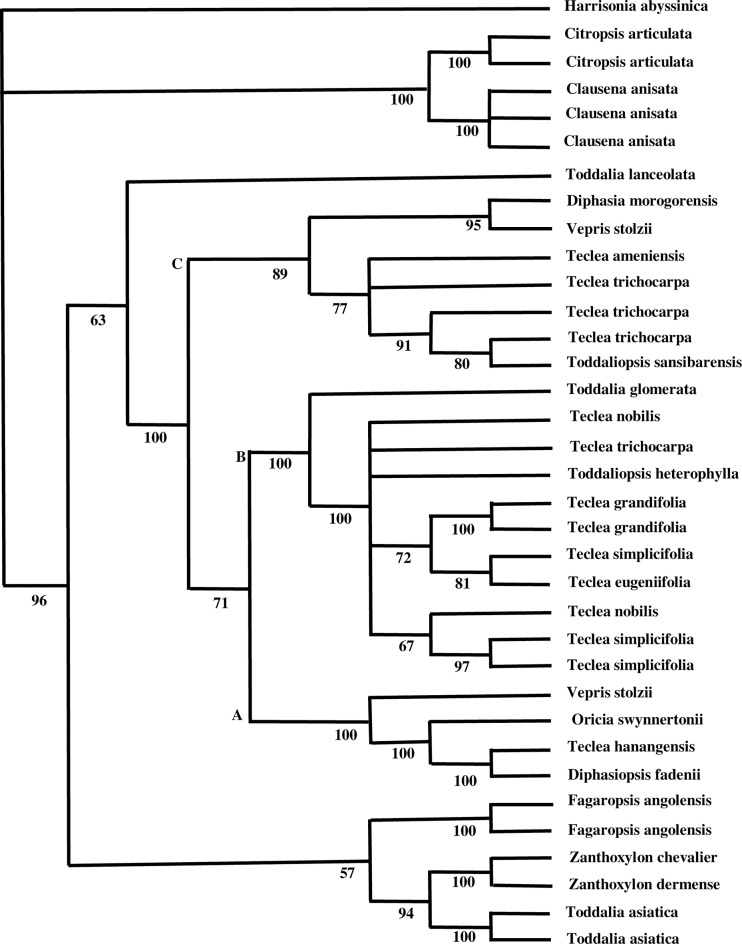
Likelihood tree using molecular data. Numbers below the branches are bootstrap values.

In the maximum likelihood analysis, the group of exemplars from *Vepris* were not monophyletic because of *Toddalia eugeniifolia* and *Toddalia glomerata* occur within the clade; Fig 4. The differences between the majority rule tree and the maximum likelihood trees was in clades B and C. In clade C, in the majority rule tree, *Teclea amanuensis* and *Teclea tricholcarpa* formed a non-supported grade to the clade containing *Toddaliopsis sansibarensis* whereas in the maximum likehood analysis they were unresolved. In clade B, in the majority rule tree, ((*Teclea nobilis*, *T*. *trichocarpa*) and *Toddaliopsis herterophylla))* formed a clade whereas in the maximum likehood analysis they were unresolved.

### Combined molecular and morphological evidence

A reduced analysis containing 18 taxa was examined using morphological characters taken from Mziray (1992) [10] taxonomic studies in Toddalieae and the ITS and *trnL* molecular datasets from above. The selection of taxa was driven from the morphology matrix contained in Mziray (1992) [10].

Multiple sequence alignment of this group resulted in a matrix of 1848 characters, of which 642 (35%) were variable and 299 (16%) were parsimony informative. The analysis recovered 81 equally optimal trees of 717 steps (CI = 0.59, RI = 0.62; Fig. not shown).

Clades A and C from the above analysis forms a polytomy consisting of four clades: 1) *Diopahsia morogorensis* and *Vepris stolzii*; 2) two species of *Teclea trichocarpa*; 3) ((*Teclea hanangensis* and *Diphasiopsis fadenii* BS 100%) *Vepris stolzii* BS 97%) and 4) *Toddaliopsis sansibarensis*. The above clade is sister to clade B which consists of ((((*Tecela simplicifolia* and *Toddalia eugeniifolia* BS 76%) *Teclea simplicifolia* BS 91%) *Teclea trichocarpa* BS 90%) *Toddalia glomerata*). At the base of the above clades was *Toddalia lanceolata* followed by a clade containing *Fagaropsis angolensis* and *Toddalia asiatica* (BS 78%).

### Phylogenetic utility of the genes (trnL-trnF and ITS)

The respective numbers of variable and potentially phylogenetically informative characters in each dataset, the consistency indices and the numbers of branches with bootstrap support above 75% can be found in Table 3. The ITS sequences produced more parsimony-informative characters when compared with the *trnL-trnF*. The ITS gene also had the highest number of resolved branches at or above 75% bootstrap support when compared with the *trnL-trnF*. The combined parsimony analysis had 20 nodes at or above 75% bootstrap support whereas the *trnL-trnF* had 3 nodes and the ITS gene had 9 nodes. There was no correlation between the increase of the CI and RI values and the increase or decrease in the number of informative characters.

**Table 3:** Genetic statistics for genes and regions utilized in the individual analyses, and in the combined Molecular datasets.

Results	*trnL*	ITS	molecular
Gaps	15	15	30
Range of Gaps	1–13	1–18	1–18
Excluded	None	None	None
Length	201	895	1134
Informative characters	120	323	443
Variable characters	241	441	682
Trees	6087	1435	54
CI (consistency index)	74	65	64
RI (retention index)	88	80	80
BB (branch and bound) above 75%	3	9	20

## Discussion

### Monophyly of *Vepris* and its closest relatives

The assembly of a larger “trnL-F” dataset including 78 taxa of Rutaceae was completed to determine the outgroup relationship of *Vepris*. Based on this analysis 19 species formed a supported clade (BS 70%) including *Diphasia*, *Diphasiopsis*, *Teclea*, *Toddalia*, *Toddaliopsis*, and *Vepris*. The genus *Acronychia*, once grouped with the above taxa, form a clade with members of *Baurella* (= *Sarcomelicope*), *Correa*, *Diplolaena*, *Eriostemon*, *Flindersia*, *Lunasia*, *Melicope*, *Phebalium*, *Pilocarpus*, and *Sarcomelicope*. At the base of these clades were member of Aurantioideae, Rutoideae and Cneoroideae. We used the genus *Harrisonia* in the Cneorioideae as the outgroup for the combined molecular analysis.

### Circumscription of *Vepris*

Both independent molecular and combined analysis of the molecular and morphological data supported that fact that the *Vepris* should be merged with other Rutaceae genera, as previously postulated by Mziray (1992) [10]. The present study examined thirty-three morphological characters, including vegetative, floral, fruit and seed features. The analysis was based on 18 taxa that Mziray (1992) [10] had included in his revision and *Acronychia* was used as the outgroup because morphological features were readily available. Only four characters provided unequivocal synapomorphies for several clades. The occurance of prickles and scrambling habit were two unequivocal synapomorphies defining *Toddalia asiatica*, while a stony endocarp feature grouped *Toddalia asiatica* and *Fagaropsis angolensis*. Species of *Telcea simplicifolia* and *Toddalia eugeniifolia* were grouped based on the unequivocal synapomorphies of unifoliolate leaf type and dominant number of leaflets. *Vepris drummondii* also shares these features but it was not included in this study and must be further examined.

Mziray (1992) [10] stated that it is rather common to find the traditional morphological characters used to be inconsistent within a species or even in the same specimen. In addition, he stated that the variation within *Diphasia* makes the distinction among *Diphasia*, *Teclea* and *Vepris* very unclear. He goes on to state that his study did not reveal other distinguishing characters with enough stability to maintain all the genera. He found that *Vepris*, *Toddaliopsis*, *Araliopsis*, *Diphasiopsis*, *Teclea*, *Oricia* and *Diphasia* formed a monophyletic group whereas *Toddalia* asiatica and *Fagaropsis* form a clade at the base of the tree. This was also evident in Fig 2 where the above genera were intermixed. Furthermore, taxa which have more than one species such as *Vepris stozii*, *Teclea simplicifolia*, *Teclea trichocarpa*, and *Teclea nobilis* did not group together (Fig 2). Most characters within these genera contain a great amount of overlap in intrageneric and intraspecific variation. For example, the leaf morphology of *Vepris* and *Teclea* contain overlap, whereas some characters (mature seeds and both stages of the hermaphrodite flowers) are difficult to interpret because of the limited amount of material available. All specimens were personally keyed out by the author, but this did not eliminate the confusion. Just as Verdoom (1926) discovered the lack of material at all stages made the task of defining satisfactory diagnostic characters for the genera and species practically impossible. However, Verdoorn’s reliance was laid too heavily on just a few morphological characters, mainly on the number of stamens relative to petals and the number of ovary cells. These characters are very plastic and this led to the recognition of rather artificial groups which have proved to be difficult to demarcate. This interspecific variation was made clear based on the molecular dataset and the analysis (Fig 2).

Mziray (1992) [10] mentions that *Oricia* species and some species of *Diphasia* and *Diphasiopsis* seem to be allied. He believed this group shared the tendency to become pubescent especially in the inflorescence and fruit; however, he also stated that no clear consistent set of characters seem to hold the group together. We found that *Oricia*, *Teclea hanangensis*, *Diphasiopsis fadenii* and *Vepris stolzii* did form a strongly supported clade which we call Clade A (BS 99) (Fig 2).

Hartley (1975) [28], stated that members of *Bauerella* Borzi, is sometimes confused with *Acronychia* because both bear dioecious flowers and opposite leaves. In the larger *trnL-F* analysis, *Acronychia*, *Bauerella*, *Sarcomeliocope* and *Meliocope* formed a clade (BS 71). *Bauerella* is currently acknowledged as a synonym of *Sarcomeliocope*.

## Conclusions

*Vepris* should be regrouped to contain the following taxa: *Vepris*, *Toddaliopsis*, *Diphasiopsis*, *Teclea*, *Oricia*, *Diphasia*, *Toddalia glomerata*, and *Toddalia eugeniifolia*, which are considered to represent a monphyletic group. The above taxa display intrageneric and intraspecific variation. This study also confirms that *Toddalia asiatica*, *Zanthoxylon sp*. and *Fagaropsis angolensis* are the closest relatives to this group.

Based on the number of informative characters and the number of branches with supported, ITS was an excellent candidate for this study. In addition, ITS produced very few alignment difficulties within the ingroup and outgroup, and its tree topology remained consistent with that of the chloroplast gene.

The phylogenetic analysis presented here provided the first study to examine the African Rutaceae using molecular data (chloroplast and nuclear), as well as morphological data. Topics to be addressed in a future study include the use of additional material and genera for determinations of species delimitations and tribal groupings.
